# Triple intussusception involving heterotopic pancreatic tissue: a case report

**DOI:** 10.1186/1752-1947-3-134

**Published:** 2009-11-18

**Authors:** N Sautot-Vial, H Steyaert

**Affiliations:** 1 Lenval Fundation for Children, Department of Pediatric Surgery, Avenue de la Californie, 06200 Nice, France

## Abstract

**Introduction:**

Intussusception involving heterotopic pancreatic tissue is a rare condition where a portion of the bowel telescopes into an adjacent segment with intraluminal pancreatic tissue as the lead point. Cases of heterotopic pancreas are most often described in the upper intestinal tract, particularly the stomach.

**Case presentation:**

We present the case of a five-month-old boy of Caucasian ethnicity suffering acute abdominal pain and vomiting with an abdominal mass in the upper right quadrant. Work-up including ultrasound scan confirmed the intussusception. Repeated attempts at radiological reduction and two laparoscopic procedures were performed within 24 hours, which eventually led to the diagnosis of a triple intussusception.

**Conclusion:**

To our knowledge, such a case of triple intussusception involving isolated heterotopic pancreatic tissue is previously unreported.

## Introduction

Intussusception is a not uncommon surgical problem occurring in children. It is defined as a prolapse of part of the intestine into the lumen of an immediately adjoining part. In infants, this entity accounts for the most common cause of intestinal obstruction. It is often idiopathic and rarely caused by an abnormal process. A triple intussusception occurs when three separate segments telescope into the same distal segment.

The management of intussusception under laparoscopy is developing and offers effective results in many patients. In our patient, however, the diagnosis could not be made after the first laparoscopy and, as a matter of fact, only one intussuscepted loop was reduced at first. Eventually, radiological clues led us to diagnose an unusual form of intussusception.

## Case presentation

A five-month-old boy of Caucasian ethnicity presented at our pediatric emergency unit. He had suffered acute paroxysmal abdominal pain and bilious vomiting for nine hours. His general condition and vital signs were normal.

During physical examination, an abdominal mass was noted in the upper right quadrant - there was no blood in stools in the rectum. Biological examinations did not yield significant results, but an ultrasound scan revealed a target sign in the form of an opaque mass under the liver. An air enema reduction was performed and identified a loop in the transverse colon that was refluxing under air pressure. This procedure did not result in a complete reduction of the assumed intussusception even after two attempts under midazolam infusion. An ileocolic intussusception of 2 cm was diagnosed during the laparoscopic exploration and a reduction was carried out by applying gentle traction. The adjacent small intestine and the ascending colon had a normal color and the child had normal peristalsis. The procedure was finished on these reassuring signs. During the night, the boy suffered repeated episodes of vomiting and had blood in his stools. An ultrasound scan performed in the morning revealed an intussusception, a swelling of the distal small intestine and the presence of a mass in the pouch of Douglas. It was difficult to determine with confidence whether the intussusception was ileocolic or ileoileal.

A second laparoscopic procedure determined that the intussusception was not ileocolic. The cecum was in its usual location, and there was no inflammation of the appendix. The small intestine was examined more closely and an intussusception was found, located 40 cm above the ileocolic junction. A reduction attempt failed even with the help of a third forceps. A right hemitransverse abdominal incision was performed but the reduction attempt failed. Palpating the ascending colon revealed a diffuse induration with no signs of wall necrosis. The small intestine appeared normal. A transverse enterotomy was performed 5 cm before the ileocolic junction. An intussusception of a bowel segment was identified but it could not be reduced. After full exploration, a tumor of less than 15 mm was identified at the top of the first intussusception. 40 cm of ileum was resected and an end-to-end anastomosis (A-B) was performed. The postoperative course was uneventful.

**Figure 1:**
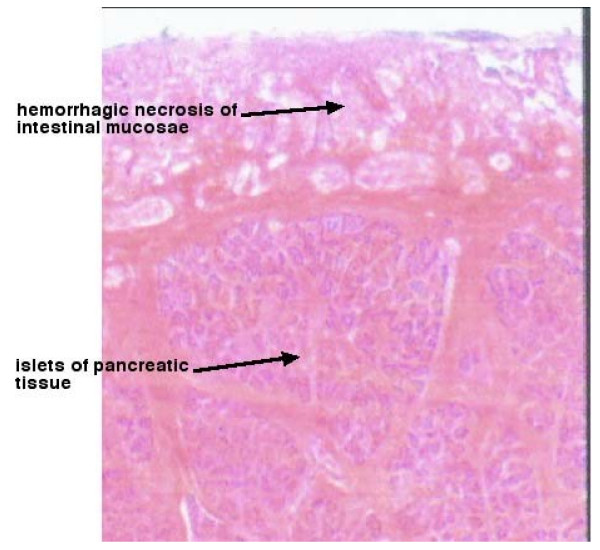
**Histologic view of the ileal lead point**.

**Figure 2:**
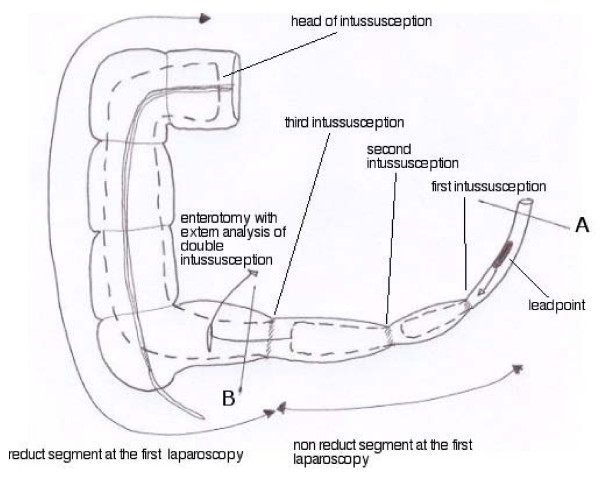
**Final study of the triple intussusception after two laparoscopic procedures and one conversion**.

Histologic examination indicated the presence of 12 mm of heterotopic pancreatic tissue (Figure 1). The first intussusception was 60 cm away from the ileocolic junction with ileal heterotopic pancreatic tissue as the lead point. The second intussusception, which formed an additional loop on the first one, was located 40 cm away from the ileocolic junction. The third intussusception completed the telescoping in the ascending colon (Figure 2).

## Discussion

This case is interesting for several reasons: (1) The triple telescoping of adjacent gut segments; (2) the isolated ileal heterotopic pancreatic tissue as lead point; (3) the failure of two laparoscopic procedures to reduce the intussusception.

We shall discuss each of these aspects in turn.

### Triple intussusception of the small bowel

In the literature, only five cases of triple intussusception have been reported both in pediatric and adult series [1,2]. They originated from benign tumors (such as a lipoma or hemangioma), hyperplasic polyp, parasitosis or heterotopic tissue (as in our patient). None of them were idiopathic. On the other hand, simple intussusception is more often idiopathic in the pediatric population. Other causes of simple intussusception are Meckel's diverticulum, angioma, digestive duplication and lymphoma. Conversely, in the adult population, the main etiology is almost exclusively of tumoral origin. The main clinical symptom remains acute abdominal pain. In all of these cases, the diagnosis of triple intussusception was made during surgery. In our patient, radiological investigations revealed only one abnormal loop in the ascending colon after an attempt at air enema reduction. In certain cases of triple intussusception, reports say that there were no abnormal signs at this stage.

### Heterotopic pancreatic tissue in the ileum

There are no cases of a triple intussusception due to isolated heterotopic pancreatic tissue described in the literature. Nevertheless, intussusceptions and double intussusceptions involving heterotopic pancreatic tissue have been reported [3-8]. The pancreas is derived from several evaginations of the wall of the duodenum. The dorsal diverticulum forms the body and tail, and the ventral portion the head of the pancreas. If an evagination is located on the bowel wall, it can form ectopic tissue anywhere in the gut [3].

Cases of heterotopic pancreatic tissue have been observed most frequently in the stomach, duodenum and jejunum. Ileal lesions are rare. The presence of heterotopic pancreatic tissue occurs in one per 500 cases of upper abdominal operations and up to 5% of autopsy cases. In the ileum, heterotopic pancreatic tissue is commonly associated with a Meckel's diverticulum. Symptoms can be associated with complications such as intestinal obstruction, bleeding from a mucosal ulcer, intussusception and obstruction of the common bile duct [4]. Half of the cases of heterotopic pancreatic tissue are asymptomatic and diagnosed incidentally.

### Laparoscopic surgery and intussusceptions

In recent years, numerous reports have described the successful management of intussusception in pediatric and adult series [9,10] and various publications relate successful laparoscopic reductions in 50 to 65% of cases. A study by Kia *et al*. [9] underlined the effectiveness of such an approach in children under 3 years and advised against its use in older patients where resection is needed more often. Exploration of the entire small bowel is easily feasible by open surgery and remains a conventional method of intussusception treatment for the majority of surgeons. On the other hand, laparoscopy is a procedure that risks silent injuries such as tears or gut perforation. In our patient, incomplete examination led to the need for re-operation after a laparoscopic attempt at reduction which missed the remaining double intussusception. Thanks to a second laparoscopic procedure performed a few hours later, we were able to diagnose this exceptional case of triple intussusception.

The literature rarely mentions triple intussusception, but references to double intussusception are not so uncommon.

## Conclusion

To our knowledge, this is the first case report of a patient with a triple intussusception involving isolated heterotopic pancreatic tissue.

## Consent

Written informed consent was obtained from the patient's parents for publication of this case report and any accompanying images. A copy of the written consent is available for review by the Editor-in-Chief of this journal.

## Competing interests

The authors declare that they have no competing interests.

## Authors' contributions

NSV was involved in conception, design, and preparation of the manuscript. HS acquired and analysed the data, and drafted the manuscript.
